# Improving Social and Personal Rhythm Dysregulation in Young and Old Adults with Bipolar Disorder: Post-Hoc Analysis of a Feasibility Randomized Controlled Trial Using Virtual Reality-Based Intervention

**DOI:** 10.3390/jcm13133786

**Published:** 2024-06-27

**Authors:** Federica Sancassiani, Alessandra Perra, Peter K. Kurotschka, Goce Kalcev, Alessia Galetti, Rosanna Zaccheddu, Aurora Locci, Federica Piludu, Lorenzo Di Natale, Valerio De Lorenzo, Michele Fornaro, Antonio Egidio Nardi, Diego Primavera

**Affiliations:** 1 Department of Medical Sciences and Public Health, University of Cagliari, 09124 Cagliari, Italy; federica.sancassiani@unica.it (F.S.); galettialessia@gmail.com (A.G.); rosannazaccheddu@gmail.com (R.Z.); auroralocci@gmail.com (A.L.); federica.piludu@outlook.it (F.P.); diego.primavera@tiscali.it (D.P.); 2 Department of General Practice, University Hospital Würzburg, 12459 Würzburg, Germany; kurotschka_p@ukw.de; 3 The National Alliance for Neuromuscular Diseases and Neuroscience GANGLION Skopje, 1000 Skopje, North Macedonia; gocekalcev@yahoo.com; 4 IDEGO Digital Psychology Society, 00197 Rome, Italy; lorenzodinatale86@gmail.com; 5 CEREBRUM VR, 00197 Rome, Italy; valerio.delorenzo@gmail.com; 6 Department of Systems Medicine, University of Tor Vergata, 00133 Rome, Italy; 7 Section of Psychiatry, Department of Neuroscience, Reproductive Science and Odontostomatology, Federico II University of Naples, 80138 Naples, Italy; dott.fornaro@gmail.com; 8 Institute of Psychiatry, Federal University of Rio de Janeiro, Rio de Janeiro 21941-901, Brazil; antonioenardi@gmail.com

**Keywords:** social and personal rhythm, bipolar disorder, COVID-19, cognitive remediation, virtual reality, advanced technology laboratory

## Abstract

**Introduction:** Rehabilitative interventions employing technology play a crucial role in bipolar disorder (BD) treatment. The study aims to appraise the virtual reality (VR)-based cognitive remediation (CR) and the interpersonal rhythm approaches to treatment outcomes of BD across different age groups. **Methods:** Post-hoc analysis of a 12-week randomizedcontrolled cross-over feasibility trial involving people with mood disorders (BD, DSM-IV) aged 18–75 years old: thirty-nine exposed to the experimental VR-based CR vs 25 waiting list controls. People with BD relapse, epilepsy or severe eye diseases (due to the potential VR risks exposure) were excluded. Biological Rhythms Interview of Assessment in Neuropsychiatry (BRIAN) was used to measure the outcome. **Results:** Cases and controls did not statistically significantly differ in age and sex distributions. Personal rhythm scores improved over the study follow-up in the experimental vs the control group (APC = 8.7%; F = 111.9; *p* < 0.0001), both in young (18–45 years) (APC = 5.5%; F = 70.46; *p* < 0.0001) and, to a lesser extent, older (>46 years) adults (APC = 10.5%; F = 12.110; *p* = 0.002). **Conclusions:** This study observed improved synchronization of personal and social rhythms in individuals with BD after a virtual reality cognitive remediation intervention, particularly in social activity, daily activities, and chronotype, with greater benefits in the younger population.

## 1. Introduction

The dysregulation of circadian biorhythms and related social rhythms (such as eating, sleeping, and engaging in social activities) is currently considered a fundamental component of bipolar disorders (BD) [1,2]. Various factors influence circadian biorhythms and related social rhythms. Lockdowns during the pandemic significantly disrupt these rhythms [3,4], particularly affecting BD [5,6,7]. Interestingly, older adults showed greater resilience in maintaining stable rhythms [8], possibly explaining lower mood disorder rates observed in this group [9,10]. Another important factor that influences rhythms is light. Light exposure, crucial for synchronizing rhythms, is disrupted during lockdowns, due to the increasing of artificial light that affects melatonin secretion and sleep [11,12,13]. Increased use of devices like e-tablets exacerbates light pollution [14,15], a trend likely to persist in modern society [16,17].

Recent observations have identified a specific syndrome characterized by the abovementioned components, the “syndrome of dysregulation of social rhythms” (DYMERS). This syndrome, believed to be closely linked to stressful conditions [18,19], could serve as a common vulnerability factor triggering various disorders due to individual risk factors. Particularly in individuals genetically predisposed to hyperactivity [20,21,22,23,24], DYMERS may act as a trigger for the onset of BD or bipolar spectrum syndromes [20,21,25]. From this point of view, interventions to regulate social rhythms are promising for BD prevention and relapse management. Cognitive remediation, including virtual reality (VR) approaches, shows potential in enhancing cognitive processes and daily functioning that targets personal recovery outcomes and, consequently, personal and social functioning [26]. Various methodologies exist, ranging from traditional approaches (paper-and-pencil and computerized) to those using technological innovations such as virtual reality [27,28]. Virtual reality-based cognitive remediation has shown significant advancements, making interventions more enjoyable, engaging, and effective than traditional methods [29]. Given the importance of synchronizing personal rhythms in BD, analyzing the role of rehabilitative interventions is essential. This study builds on previous research [30], which demonstrated that VR-based cognitive remediation is feasible and potentially effective in synchronizing biological and social rhythms, along with improving cognitive functions, depressive symptoms, anxiety, quality of life, global functioning, and alexithymia in BD patients.

For these reasons, this work aims to conduct a secondary analysis of the database of the previously described study to investigate the specific dimensions of personal rhythms that have improved significantly post-intervention in the experimental group compared to the control group. Specifically, we aim to compare different age groups and verify whether the improvement occurs in the younger population (<45 years) and the older population, including individuals over 65.

The hypothesis is that a virtual reality-based cognitive remediation intervention using an integrated and recovery-oriented approach can yield positive effects not only on cognitive functions but also on social and biological rhythms, which are important components in the treatment of BD. Additionally, it is hypothesized that there will be a difference in the improvement of rhythms between younger and older populations, as well as differences in the specific dimensions of rhythms. Older individuals seem to demonstrate more stable rhythms, making them more resistant to factors that disrupt rhythms, while young adults exhibit greater instability; hence, the latter might benefit more from an effective intervention targeting the regulation of personal rhythms.

## 2. Methods

### 2.1. Design

This study is a secondary analysis of a randomized controlled cross-over feasibility trial, registered on Clinical.Trial.gov (NCT05070065, 21 July 2021) [30,31]. This trial adhered to the CONSORT extension guidelines for feasibility studies [32].

### 2.2. Study Sample

The sample included people diagnosed with mood disorder (BD, DSM-IV) who were recruited from those undergoing treatment at the Consultation Psychiatry and Psychosomatic Center of the Hospital “San Giovanni di Dio” (University Hospital of Cagliari). Inclusion criteria were ages 18–75, a diagnosis of BD made by a psychiatrist according to DSM-IV criteria [33], and inclusion of all genders. Exclusion criteria were the presence of current manic/depressive episodes (due to the inability to adhere to interventions), serious eye diseases or concurrent epilepsy (as the e-stimulation of virtual reality could cause some risks), as well as participants who did not provide informed consent before the intervention began. After the eligibility phase, participants were randomly assigned into two arms groups (experimental and control group), which were carried out using an online computer-generated list with an allocation ratio of 1:1. Randomization was conducted by a biometrician blinded to the participants’ identities and not involved in the following study phases. Both participants and researchers conducting participant evaluations were blinded to the type of intervention (experimental or control). The control group was on an inactive waiting list, receiving treatment as usual (pharmacotherapy and psychiatric visits). 

### 2.3. Experimental Intervention

People in the experimental group were involved in a fully immersive Virtual Reality. Cognitive Remediation intervention applying the “CEREBRUM—Cognitive Rehabilitation” software version 3.0.1, developed by the company Cerebrum VR Society (Rome, Italy). Previously published papers have detailed the software and session methodology [30,31]. In summary, CEREBRUM features virtual scenarios that replicate everyday life, including home and urban environments. It offers 52 exercises of increasing difficulty: 20 exercises are within the Attention and Working Memory Module, 22 exercises are part of the Memory and Learning Module, and 10 exercises are included in the Cognitive Estimates Module.

The increasing difficulty levels enable clinicians to adjust the intervention’s difficulty based on participants’ functional performance and specific abilities. Within this framework, the learning context becomes stimulating as exercises are tailored to participants’ levels in everyday life contexts. The intervention consisted of 24 sessions, each lasting 45 min (two weekly sessions for three months). Specifically, each session starts with an introduction that incorporates mindfulness techniques to support emotional integration into the cognitive learning process, psychoeducation on cognitive functions to boost awareness and self-monitoring, virtual reality-based training, and generalization activities to help apply learned strategies in the individual’s daily life according to their needs. During the training portion of each session, users complete 2 to 3 virtual reality exercises: 1 from the Attention and Working Memory Module, 1 from the Memory and Learning Module, and 1 from the Cognitive Estimates Module, progressing through all exercises. The exercises are performed in the order they are presented in the app, ensuring comprehensive completion over time.

The sessions were replicable and designed based on a human-centered approach for complex interventions [34] and a recovery-oriented social inclusion intervention [35,36]. This means that the techniques used are multidimensional, ranging from welcoming with relaxation techniques to psychoeducation, and training in real-life contexts through generalization strategies related to personal recovery goals, in line with the theoretical framework [37,38,39]. The evolutionary framework of social determinants of health was used as a model for developing the rehabilitation intervention methodology, with the primary objective being the achievement of personal goals and, consequently, an improvement in the various dimensions involved in BD [40,41,42].

### 2.4. Outcome and Study Tools

This publication explores the relevant findings regarding regulating biological rhythms, which are considered a secondary outcome in the trial protocol. The outcome measure is the score obtained on the Biological Rhythms Interview of Assessment in Neuropsychiatry (BRIAN) [43] administered in its validated Italian version [44]. This tool comprises 21 items, allowing us to assess five areas of rhythms: sleep, activities, social interactions, meal rhythms, and chronotype (i.e., predominant rhythm). All items utilize a four-point scale, from 1 = not at all to 4 = often; higher scores indicated greater misalignment in the specific social or personal rhythm. The scale has been translated into several languages [45,46].

### 2.5. Statistical Analysis

The change over time in the BRIAN score was calculated as the difference in the mean score ± standard deviation using one-way ANOVA statistics for repeated measures. The average percentage change (APC) was calculated by determining the difference in percentage change (in terms of gain) between the experimental group and the control group.

Subsequently, differences in score change T0 vs T1 between groups were assessed using one-way ANOVA statistics. Comparison for nominal variables was conducted using chi-square tests. To assess the normality of the distribution of the different variables, the Kolmogorov–Smirnov Test of Normality was utilized. These analyses were used to study how the differences evolved over time and across various subgroups, allowing for the assessment of the magnitude of the changes.

All analyses were performed using SPSS software (version 28.0.1.0., IBM, Armonk, NY, USA), with a *p*-value < 0.05 considered statistically significant.

## 3. Results

**Figure 1:**
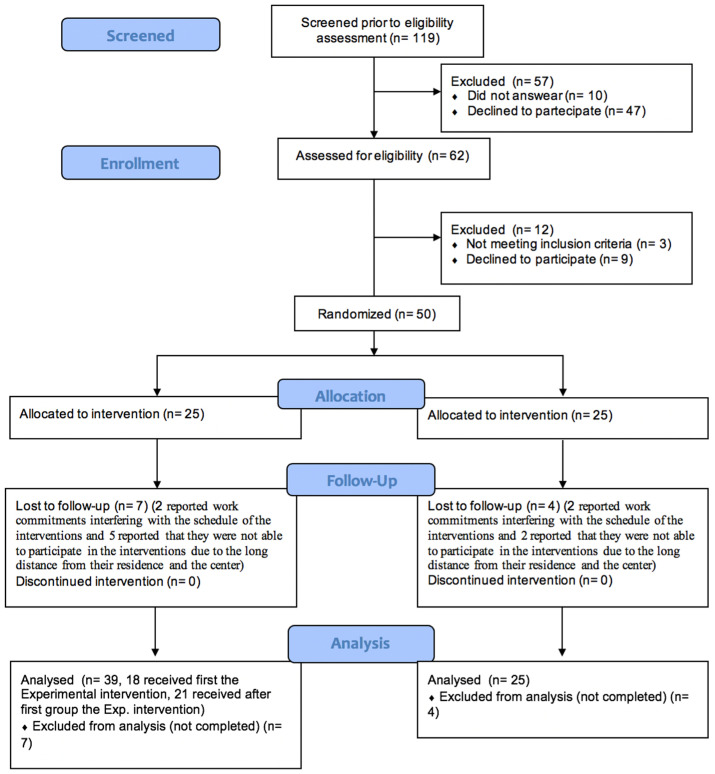
CONSORT flow diagram extension for feasibility study.

The final sample comprised 39 individuals in the experimental harm (after dropouts at follow-up) and 25 in the control group (Figure 1). The mean age in the overall final sample was 47.23 ± 13.37 years, 67% were females. In the experimental group the mean age was 47.51 ± 13.52 years, 64% were females; in the control group the mean age was 46.28 ± 13.40 years, 72% were females. No statistically significant differences were found between the experimental and control groups by age (F = 1.63; df = 0.127; *p* = 0.723) and sex (1df = 0.431; *p* = 0.512). Regarding the subgroups, the older sample (>45 years) included 17 individuals in the experimental group and 11 in the control group; the younger sample (<46 years) included 22 individuals in the experimental group and 14 in the control group.

The study confirmed the differences by time and group in the BRIAN score, with an improvement in the experimental group subjected to the virtual reality treatment compared to the control group, as shown in Table 1 (APC = 8,7%; F = 111.9; *p* < 0.0001); our study highlighted that although the difference between the experimental and control groups was greater in young adults (APC = 10.5%; F = 70.46; *p* < 0.0001), there was conclusive efficacy of the treatment in synchronizing the rhythms even in older adults (APC = 5.5%; F = 12.110; *p* = 0.002).

As shown in Table 2, the treatment with virtual reality is associated with an improvement in the score of the BRIAN activity (F = 112.2; *p* < 0.0001), social (F = 93.74; *p* < 0.0001), and chronotype subscales (F = 84.35; *p* < 0.0001). However, the subscales relating to sleep and meal patterns do not improve.

## 4. Discussion

There was an evident improvement in the experimental group to which the virtual reality treatment was administered compared to the control group, showing the improvement was greater in young adults. Still, there was a clear efficacy of the treatment in synchronizing the rhythms even in older adults in who were found to have more stable rhythms and, therefore, would be more resistant to changes [8].

The treatment with virtual reality was associated with an improvement in the score in the experimental group compared to the control group in the BRIAN subscales of activity, social, and chronotype rhythms. However, the subscales relating to sleep and meal patterns did not improve. The results confirmed the hypothesis, suggesting that there are differences in the specific dimensions of rhythms, particularly improved social activities and chronotype rhythms and not sleep rhythms, and in the subgroups of younger and older individuals.

The virtual reality experiment tested in this study [30] was specifically designed and conceived to improve cognitive performance in people suffering from BD given the risk of cognitive impairment in the long-time course of this disorder [47,48,49]. However, the presentation of the first results [30] brought to attention the improvement in the synchronization of social and personal rhythms due to the virtual reality treatment. It is known that the dysregulation of personal and social rhythms, linked to biological chrono rhythms, is an important element in the onset [50,51], worsening, and relapses [52,53,54] of BD. Additionally, based on the hypotheses of this study, we aimed to investigate whether there were differences in the specific dimensions of rhythms and in the subgroups of younger and older individuals. For this reason, it was useful to delve deeper into this secondary analysis.

These data demonstrate that the intervention with virtual reality (which proposes exercises based on activities of daily life) improves the aspects linked to chronotypes of social interactions and common life activities but not specifically the aspects more deeply linked to biorhythms such as sleep [55,56] and the rhythm of meals [57,58]. Previous studies have observed that cognitive remediation in BD improved cognitive abilities and other outcomes, such as the overall quality of life [59]. The homework exercises of cognitive remediation, thanks to offering a daily structure and opportunities for exchanging coping strategies, effectively enhanced social changes and inclusion [59]. These aspects are probably amplified through the application of virtual reality; this tool presents pleasant and stimulating aspects that can well counteract the vulnerability to boredom of people with BD [60]. The effect of better synchronization in daily activities and social interaction could be due to greater ease derived from exercises with virtual reality. From this point of view, it is possible that in people with previous episodes of hypomania/mania and mild acquired cognitive impairment, the relapse may also be a function of “acceleration to deny the difficulties”. Anyway, the improvement highlighted in the score relating to the synchronization of social rhythms and daily activities, although it is not such as to affect the rhythms of sleep and eating, is associated with an improvement (or rather with a non-worsening), in comparison with the control group, in the chronotype (i.e., morning energy), which affects, however, a central aspect of BD [61]. These results are also consistent with other studies that have examined the impact of cognitive rehabilitation and psychoeducation interventions on the improvement of cognitive processes, symptoms, and biological and social rhythms using the same instrument, the BRIAN instrument [62,63].

### 4.1. Limitations

The main limitations of the present study are related to the sample size and Berkson’s bias. Berkson’s bias occurs particularly in hospital-based studies where the sample is taken from patients seeking treatment at a hospital or clinic. In such settings, patients are more likely to have multiple conditions compared to the general population, which can create a spurious association or obscure a real association between the risk factor and the disease. In particular, the sample size is too small to demonstrate the effectiveness of these results, and additionally, an intention-to-treat analysis was not performed.

### 4.2. Implication for Research

Regarding research implications, it is important that these findings be confirmed in studies with larger sample sizes capable of demonstrating their effectiveness. This study also provides interesting insights into the differences in improvement among various age subgroups, which can help understand the influencing factors.

## 5. Conclusions

This study found that virtual reality cognitive remediation improved the synchronization of personal and social rhythms in people with BD, particularly in social activity, daily activities, and chronotype, with greater effects in younger participants. These findings highlight the importance of rhythm synchronization in BD treatment, suggesting the need for developing and evaluating more specific virtual reality tools. Clinically, this supports using virtual reality for cognitive remediation with integrated, recovery-oriented approaches in both young and older individuals with BD.

## Figures and Tables

**Table 1:** BRIAN total score comparison by time (T0 vs T1) and experimental and control groups (EG vs CG) and by subgroups divided by age (young adults (<46) and older adults (>45)) with APC and one way-repeated measures ANOVA (F, *p* value).

BRIAN Total Score	TO Mean±SD Normality Test	T1 Mean ± SD Normality Test	T0 vs T1 Mean Difference ± SD
EG (N = 39)	49.51 ± 12.70 *p* = 0.799	47.35 ± 12.14 *p* = 0.656	−2.16 ± 1.02
CG (N = 25)	48.12 ± 12.29 *p* = 0.648	50.24 ± 12.13 *p* = 0.893	+2.12 ± 2.19
APC = 8.7%; F = 111.9; *p* < 0.0001			
EG > 45 (N = 17)	49.05 ± 13.52 *p* = 0.867	47.64 ± 11.70 *p* = 0.895	−1.41 ± 1.53
CG > 45 (N = 11)	48.36 ± 13.99 *p* = 0.367	49.63 ± 12.86 *p* = 0.854	+1.27 ± 2.56
APC = 5.5%; F = 12.110; *p* = 0.002			
EG < 46 (N = 22)	49.86 ± 12.02 *p* = 0.760	47.13 ± 12.54 *p* = 0.749	−2.37 ± 1.44
CG < 46 (N = 14)	47.92 ± 10.77 *p* = 0.777	50.71 ± 11.51 *p* = 0.828	+2.79 ± 2.26
APC = 10.5%; F = 70.46; *p* < 0.0001			

**Table 2:** BRIAN score at different components comparison by time (T0 vs T1) and groups experimental group (EG) vs controls group (CG).

BRIAN Components	T0	T1	T0 vs T1
EG (N39) Sleep	12.74 ± 4.27	12.41 ± 3.63	−0.31 ± 0.49
CG (25) Sleep	12.64 ± 3.65	12.28 ± 3.83	−0.36 ± 0.58
			F = 0.137; *p* = 0.712
EG (N39) Activities	13.79 ± 3.99	12.41 ± 3.63	−1.38 ± 0.98
CG (25) Activities	12.24 ± 4.15	13.28 ± 4.34	+1.04 ± 0.73
			F = 112.2; *p* < 0.0001
EG (N39) Social	8.61 ± 3.04	7.89 ± 2.68	−0.72 ± 0.45
CG (25) Social	8.60 ± 2.57	9.12 ± 2.90	0.52 ± 0.57
			F = 93.74; *p* < 0.0001
EG (N39) Feeding rhythm	8.15 ± 3.10	8.25 ± 3.63	+0.10 ± 0.52
CG (25) Feeding rhythm	8.6 ± 3.47	8.84 ± 3.24	+0.24 ± 0.38
			F = 0.134; *p* = 0.250
EG (N39) Chronotype	6.20 ± 2.02	6.10 ± 2.21	−0.10 ± 0.25
CG (25) Chronotype	6.04 ± 2.19	6.72 ± 1.92	+0.68 ± 0.43
			F = 84.35; *p* < 0.0001

## Data Availability

Data are contained within the article.
